# Renal Denervation Influences Angiotensin II Types 1 and 2 Receptors

**DOI:** 10.1155/2022/8731357

**Published:** 2022-10-10

**Authors:** Hajaralsadat Hosseini-Dastgerdi, Fatemeh Kharazmi, Ali-Asghar Pourshanazari, Mehdi Nematbakhsh

**Affiliations:** Water & Electrolytes Research Center and Department of Physiology, Isfahan University of Medical Sciences, Isfahan, Iran

## Abstract

The sympathetic and renin-angiotensin systems (RAS) are two critical regulatory systems in the kidney which affect renal hemodynamics and function. These two systems interact with each other so that angiotensin II (Ang II) has the presynaptic effect on the norepinephrine secretion. Another aspect of this interaction is that the sympathetic nervous system affects the function and expression of local RAS receptors, mainly Ang II receptors. Therefore, in many pathological conditions associated with an increased renal sympathetic tone, these receptors' expression changes and renal denervation can normalize these changes and improve the diseases. It seems that the renal sympathectomy can alter Ang II receptors expression and the distribution of RAS receptors in the kidneys, which influence renal functions.

## 1. Introduction

The renal sympathetic nerve is the primary vasoconstriction system that affects blood pressure [1]. The RAS is primarily involved in controlling blood pressure and body fluids [2]. However, there is a solitarily and localized RAS in the kidneys, which work under the influence of the renal sympathetic system [3]. In the absence of kidney sympathetic in normal conditions, the density and number of Ang II receptors in the kidney increase, so the renal sympathetic nerve regulates the expression and function of local RAS receptors [4]. On the other hand, increasing renal sympathetic activity under various mechanisms leads to vasoconstriction, diminution of renal blood flow (RBF), and reduction of glomerular filtration rate (GFR), followed by renin release, formation of Ang II, reduction of urinary sodium excretion, and increase of systemic blood pressure [5, 6]. One mechanism that has been less studied is the change in Ang II receptors, while renal denervation may improve the pathological condition by shifting changes of Ang II receptors to normal [7–9]. The relationship between sympathetic activity and Ang II receptors expression in the kidney is a scientific challenge. In this review, various popular databases such as PubMed, Google Scholar, and Scopus were considered for related information.

### 1.1. Renal Nerves

The kidneys have afferent and efferent nerve fibers [10]. The afferent nerves originate from the proximal urethra, around the great vessels, adventitia, and smooth muscle layer in the kidney pelvis [11]. The peripheral dispensation of the afferent neurons creates a perfect stretch receptor that covers the renal pelvic wall. Therefore, in response to chemical (inflammatory mediators) and mechanical (increasing pelvic pressure) stimuli and pain (kidney calculi), afferent or sensory nerve fibers send impulses to the central nervous system (CNS) [11, 12]. The renal efferent nerves are mainly adrenergic [13], transmit nerve impulses from the CNS to the kidneys, and affect renal functions [1, 10]. So, by releasing norepinephrine, the efferent fibers cause vasoconstriction in renal arteries, reabsorption of water and sodium in the epithelial cells of the renal tubules, and release of renin from the juxtaglomerular cells [1]. Afferent and efferent nerves are distributed separately in the renal pelvis [14].

### 1.2. RAS and Renal Functions

RAS mainly controls sodium homeostasis and blood pressure [2], and the kidneys also have an intrarenal RAS [15]. Ang II is the main component of RAS with two receptors of type 1 and type 2 (AT1R and AT2R) [16]. Angiotensin I is generated from renin, and then, ACE metabolizes Ang I to Ang II [17]. ACE2 metabolizes Ang II to angiotensin 1–7 (Ang-(1–7)) [18]. Ang-(1–7) functions by binding to Mas receptor (Mas R), which opposes AT1R [19]. Also, Ang-(1–7) through Mas *R* has various direct actions within the kidney [20].

The AT1R is a member of the G-protein-coupled receptor [21]. AT1R in rodents has two subtypes including AT1aR and AT1bR. These subgroups exist in different tissues in different proportions, although their signaling and ligand binding mechanisms are almost the same. Cardiovascular tissues mostly express AT1aR, while AT1bR is predominant in tissues such as adrenal glands and pituitary gland [22, 23]. Although studies state that AT1R does not have such isoforms in humans [23, 24], one study showed that AT1bR is a new subtype of human AT1R that is expressed in the lung, placenta, and liver and is different from AT1aR in terms of tissue distribution [25]. In the kidney, AT1R is distributed in glomerular mesenchymal cells, proximal and distal tubular epithelia, medullary interstitial cells, and renal vessels [26–29]. The AT1R increases the activity of the cyclooxygenase system through phospholipases *D* and A2 and causes decrease in cyclic adenosine monophosphate (cAMP) and increase in intracellular calcium [30–32]. cAMP causes excitatory regulation of renin from juxtaglomerular cells (JG) [33]. Adenylate cyclase (AC) produces cAMP, which activates protein kinase A (PKA). Free catalytic subunits of PKA phosphorylate the transcription factor cAMP responsive element binding (CREB) protein in the nucleus, which activates the renin gene [34]. So, Ang II through AT1R inhibits JG renin synthesis (shortened negative feedback) [26]. Unlike JG cells, Ang II increases renin secretion in collecting duct principal cells through AT1R [35]. This regulation is dependent on PKC-a (calcium-dependent PKC), which leads to an increase in cAMP production through adenylate cyclase 6 (AC6) and thus activates the PKA/CREB pathway [35]. Also, Ang II increases renal sodium uptake [36], diminishes RBF, and increases the efferent arterial resistance and glomerular filtration pressure [37] by stimulating AT1R. It should be noted that long-term high level of circulating Ang II induces renal oxidative stress by stimulating the transcription of various NADPH oxidase subunits, including Nox2 and Nox4 through AT1R activation [38]. Other effects of AT1R are unrelated to the tubular and hemodynamic impact on the kidneys [39]. For example, the stimulation of AT1R causes hyperplasia and hypertrophy of vascular cells [39], production of reactive oxygen species [40], induction of inflammation [41], thrombosis [26], and fibrosis [26].

AT2R is distributed in renal vessels, glomeruli, juxtaglomerular apparatus, tubules, arteries, and renal capsules [26, 42], and this receptor is involved in the regulation of renal hemodynamic and tubular functions [43]. The stimulation of AT2R in the afferent arterioles has a vasodilatory effect [44] and in the renal tubules causes sodium excretion and pressure natriuresis [45]. In kidney, the preferred ligand of AT2R is Ang III (a metabolite of Ang II), which is essential for processes mediated by AT2R, including natriuresis [46–48]. Also, the stimulation of AT2R inhibits bicarbonate reabsorption in the proximal tube, independent of hemodynamic alterations (directly) [26, 49]. It is shown that AT2R stimulation boosts medullary and cortical bradykinin-nitric oxide (NO)-cGMP cascade. AT2R increases PGE2 through the bradykinin-NO-cGMP pathway and leads to an increase in its conversion to PGF2*α*, which acts as an inhibitor of renin secretion [47, 48]. Renal levels of bradykinin, NO, and cGMP decrease when AT2R is blocked by its antagonist (PD123319) [50]. Although AT2R, like AT1R, is a member of the G-protein-coupled receptor family, it has various signaling mechanisms, tissue expressions, and functions [26].

Mas *R* is a GPCR [51] that is expressed in the kidney in afferent arteries, mesangial cells, and the apical surface of the tubular epithelium, and Ang-(1–7) regulates renal hemodynamics and tubuloglomerular function through it [52]. The effects of the Ang-(1–7)/MasR pathway are opposite to the effects of the Ang II/AT1R pathway and include vasodilatory [53], antifibrosis [54], and anti-inflammatory [55] effects in various tissues and natriuresis through inhibition of the Na + -K + ATPase pump in kidney tubules [56]. Ang-(1–7) increases the expression of the glomerular renin gene, which can be due to the disinhibition of the AT1R negative feedback cycle caused by AT1R downregulation [47]. The Ang-(1–7)/MasR signaling pathway through the phosphoinositide 3-kinase (PI3K)/Akt/endothelial nitric oxide synthase (eNOS) pathway causes the release of NO from endothelial cells [57].

It can be summarized that renal hemodynamics alteration and its functions are influenced by RAS and its components and will alter depending on the RAS and its component's activities.

### 1.3. RAS and Renal Sympathetic Nerves

Renal function is controlled by RAS and renal sympathetic nerves [58]. The effects of sympathetic nerves on the kidneys may diminish by inhibiting ACE or AT1R blocking (suppressing RAS activity). It is reported that intrarenal Ang II administration has a slight renal effect in denervated kidneys [59]. Regarding the interactions between RAS and sympathetic kidney nerves, it has been suggested that a certain intensity of RAS activity is needed to modulate its presynaptic action, releasing norepinephrine from renal sympathetic nerve terminals [60]. Norepinephrine stimulates *β*1 adrenergic receptors in juxtaglomerular cells and releases renin. Ang II formed by renin activity can have a presynaptic or postsynaptic action on AT1R and AT2R in the tubules [59]. The water and chloride reabsorptions in the proximal tubule increase by Ang II; however, the water and chloride reabsorptions reduce by ≈75% after denervation. This finding suggests that in absence of renal sympathetic effect, Ang II's absolute function via Ang II receptors in the proximal tubules is about 25% [59].

Resulting from this interaction, the absence of renal sympathetic compensatory changes the Ang II receptors [4]. Both AT1R and AT2R interact with Ang II and the renal sympathetic nerves in regulating renin release. Sub-acute renal denervation with cessation of renal adrenergic activity leads to a decrease in the local concentration of Ang II due to a reduction in the stimulus of endogenous renin production, which leads to an increase in the number and density of renal Ang II receptors [4, 61, 62]. The regulation of Ang II receptors alone is inadequate to compensate for the deficiency of renal adrenergic input [4]. Therefore, arterial resistance in denervated kidneys decreases in plasma expanded conditions compared to the innervated kidneys [63, 64]. In terms of a crosstalk link between renal AT1R and *α*1-adrenoceptor subtypes, an increase in AT1R in the renal vasculature in the denervated rat kidneys revealed a possible interaction between the sympathetic nerve and RAS [65]. Pelayo and Blantz examined the importance of the activity of renal adrenergic fibers in the indirect regulation of Ang II receptors, and they stated that the most likely mediating link is the receptor regulatory response to changes in rate or the concentration of Ang II production in the kidney due to nerve absence [4, 66]. In this regard, Wilkes et al. evaluated the number of Ang II receptors seven days after unilateral renal denervation in rats, and they found that the glomerulus of denervated kidneys contained 50% more receptors than controls [67]. Moreover, B Sahlgren et al. demonstrated that in both hypertension and normotensive rats, left kidney denervation led to a 50% increase in glomerular Ang II receptor density in the denervated kidney 8–10 days following unilateral renal denervation [61]. In addition, the variation in the number of Ang II receptors in the denervated and innervated kidneys was eliminated by the pharmacological intervention of RAS that blocks the formation of Ang II (using enalapril) or increases circulating Ang II (pharmacological dose of Ang II) [67]. So, local RAS activity at the tissue level is principal in regulating the number of Ang II receptors [67]. Also, it should be noted that a study showed that unlike AT1 receptors in the glomerulus, proximal tubule AT1Rs be positively regulated by Ang II [68]. Therefore, it seems that renal denervation by reducing the Ang II concentration causes a decrease in Ang II receptors in the proximal tubule. However, the effects of renal denervation on tubule Ang II receptors in different areas of the kidney under normal conditions are ambiguous and there are no data to support or reject the latter hypothesis.

Zuzana Honetschlägerová et al. showed that aorto-caval fistula Ren-2 transgenic rats show an exaggerated RBF responsiveness to intrarenal Ang II. This responsiveness increased following renal denervation (RDN). One possibility that these authors initially stated was that the increase in vascular responsiveness induced by RDN depends on an increase in the density or affinity of AT1R and a decrease in the density or affinity of AT2R. However, they ultimately concluded that the exaggerated RBF response to Ang II could not simply be attributed to an increase in the number or affinity of AT1R before or after RDN [69]. In this regard, another study reported that there is no change in Ang II receptor binding affinity after subacute denervation, and an increase in vascular response was observed after denervation, indicating that renal denervation creates a stimulus to increase the number of Ang II receptors [4]. Accordingly, it seems that renal sympathectomy causes alteration in the density, affinity, or number of renal Ang II receptors.

### 1.4. The Sympathetic Nervous System and Pathological Conditions

Many cardiometabolic diseases are associated with overactive sympathetic nerves, but understanding of the mechanism of these relationships is limited [5]. Renal ischemia and oxidative stress are two examples of the stimuli that cause the afferent signal in the sensory fibers of the kidney to rise. Through its modulatory effect on posterior hypothalamus activity, an increase in renal sensory afferent signals influences sympathetic outflow and impacts the kidneys as well as other strongly innervated organs including the heart and peripheral blood vessels [70]. Reduced GFR, RBF, and sodium excretion are linked to sympathetic hyperactivity, which can impact systemic blood pressure. Studies have shown the value of renal denervation as a therapeutic technique to decrease blood pressure and have emphasized the connection between renal sympathetic afferents and efferent nerves and the pathophysiology of hypertension, heart failure, and chronic kidney disease [70]. Based on these phenomena, renal sympathectomy is along with the reduction of blood pressure, and it could be applied to treat hypertension in obesity [6], heart failure [71], chronic kidney diseases [72], and insulin resistance [9, 73] in individuals whose disease is associated with increased sympathetic reflexes tone. In these conditions, local RAS receptors change due to overactive sympathetic activity [74–76]. One of the mechanisms by which renal denervation improves this condition is the normalization of local RAS receptors, as discussed in the following.

### 1.5. The Role of Renal Denervation on Renal Ang II Receptors in Pathological Conditions

A model for primary hypertension is spontaneously hypertensive rats [77]. This genetic model's etiology of hypertension is unclear [78]. However, the sympathetic nervous system (SNS) and RAS both play significant roles in the onset and maintenance of hypertension [7]. It has been shown that spontaneously hypertensive rats lacking renal nerve respond to vasoactive substances more strongly than spontaneously hypertensive rats with intact renal nerve. When compared to denervated SHR rats that did not receive losartan, the responsiveness of the renal vasoconstrictor to vasoactive substances was reduced in losartan-treated denervated SHR rats. These findings suggest that the function of AT1R in these rats' renal arteries may have increased [65]. In addition, the vasoconstriction responses to Ang II and adrenergic agonists were reduced by beta-blocker (carvedilol) in hypertensive rats, and after the elimination of renal sympathetic activity, carvedilol function was altered by increasing the sensitivity of a1-adrenoceptors and AT1R to vasoconstrictors [7]. The effect of renal denervation in this model of hypertension is to increase the sensitivity and function of AT1R to vasoconstrictors.

It should be noted that in several models of hypertension, including two kidneys one clip (2K1C) model, renal denervation can cause a continuous decrease in blood pressure [79, 80]. However, the underlying mechanisms by which renal denervation reduces blood pressure, are not fully understood [76]. In the 2K1C rat model, the Ang II receptors expression was determined in both kidneys, and the results showed a significant upregulation in Ang II receptors mRNA expression in the clipped one; however, renal denervation caused normalization of their expression in the ischemic kidneys [76].

Chronic heart failure (CHF) is characterized by sympathoexcitation, particularly in the heart and kidneys [81]. Increased renal sympathetic nerve activity causes renal vasoconstriction, decreased RBF, increased water and sodium reabsorption, and renal fibrosis [82]. These changes also increase cardiac preload and RAS activity, which ultimately leads to cardiac muscle remodeling and intensification of heart failure (HF) [81]. In CHF, the elevation of renal nerve activity can affect AT1R expression in renal vessels [8]. Due to the increased activity of renal sympathetic fibers, receptor profiles and renal cellular signaling pathways change [75]. So, the interaction between AT1R vasoconstrictor properties and AT2R vasodilator effects has shifted mainly to vasoconstriction in CHF [75]. The balance of AT2R and AT1R expressions in CHF varies, and AT1R expression is higher than AT2R [83]. This balance has been refined following renal denervation [75], and unilateral renal denervation results in a near-normal gene expression pattern, i.e., AT1R decreases and AT2R increases [83]. It is not exactly clear how renal denervation modulates AT1R and AT2R expression still, and it is possibly caused by the direct effect of the kidney nerve on the transcription or trafficking of AT1R and AT2R [84]. However, a study was also conducted by Zuzana Honetschlägerová and colleagues and showed that the effect of renal denervation on reducing HF mortality was unrelated to renal mechanisms. They showed that despite a significant decrease in blood pressure, renal denervation does not alter renal AT1R in mRen2 transgenic rats with HF [69].

On the other hand, cardiomyopathy is a disease that affects the heart muscle and can lead to HF [85]. In the animal model of cardiomyopathy, the renal denervation caused the downregulation of the Ang II/ACE/AT1R axis and the upregulation of the Ang-(1–7)/ACE2/Mas-R axis [74].

Cardiorenal syndrome is the coexistence of CHF and chronic kidney disease that exacerbate each other [86]. The SNS and RAS play an essential role in the cardiorenal cycle [87]. In a model of L-NAME (N-nitro-L-arginine methyl ester)-induced cardiorenal syndrome, the upregulation of angiotensinogen and AT1aR mRNA expression occurred in the left ventricle, downregulation was detected in the renal cortex, and these changes were refined by bilateral renal denervation [88].

Aortic regurgitation (AR) is a valvular disease resulting in the reflux of blood from the aorta to the left ventricle during diastole [89]. Various causes can affect the valve and root of the aorta and cause AR [89]. In rats with AR, SNS activity and intrarenal norepinephrine increase levels of AT1aR and angiotensinogen mRNA in renal cortical tissue and renal denervation suppresses them [90].

Polycystic kidney disease (PKD) is a common renal disease that often leads to end-stage kidney disease [91]. The disease is associated with cardiovascular complications, including hypertension and HF [92]. Evidence has shown that an overactive nervous system and intrarenal RAS are involved in its pathogenesis [93, 94]. The Lewis polycystic kidney (LPK) rat is a model of PKD with an early start of hypertension. Studies have shown that, compared to controls, this PKD model had reduced renal renin concentration and intrarenal RAS expression (including AT1aR mRNA). Pressure-dependent feedback in juxtaglomerular renin secretion is the cause of renin reduction. However, the mechanism causing the decrease in intrarenal RAS is not clearly identified [95]. Renal denervation does not impact hypertension or RAS, which may be because other pathways involved in controlling intrarenal RAS, such as the prorenin receptor, nuclear receptors, and prostaglandins, offset RDN effect. So, renal denervation may not be appropriate for reducing blood pressure in patients with PKD [95].

Renal nerves play an important role in structural damage in diabetic nephropathy. Renal denervation after the onset of diabetes reduced the progression of diabetic nephropathy [96]. In the streptozotocin (STZ)-induced diabetes model, there is a reduction in the number of Ang II glomerular sites associated with hyperfiltration [97–99]. Since pretreatment with magnesium chloride led to the breakdown of the hormone that was already bound, occupancy of the receptor could not have been the root of this problem. Plasma Ang II levels were the same in control and diabetic rats, indicating that angiotensin II did not induce a decrease in angiotensin II receptor density. In diabetic and control rats, angiotensin II infusion and ACE inhibitor, respectively, resulted in receptor downregulation and overexpression, although the receptor difference persisted [99]. Insulin replacement improves glomerular angiotensin II receptor insufficiency. The restoration of angiotensin II receptor density to normal levels in chronic diabetes may be caused by receptor upregulation by increased plasma aldosterone [99]. On the other hand, STZ-induced moderate increased AT1R in the renal collecting ducts for 20 days [100, 101]. Also, examining the animals' kidneys that have undergone hyperglycemia for a more extended period (12 weeks after STZ) shows that induction of diabetes increases AT1R expression in the cortical and outer medullary collecting duct [96]. Due to the increased urinary output in diabetes, the increase in AT1R in the collecting ducts is essential for maintaining the balance of sodium and water, which is mediated by Ang II [100]. LM Harrison-Bernard et al. assumed that increase in AT1R protein production reflects an increase in functional receptors that connect to intracellular pathways. Consequently, it is anticipated that Ang II-dependent effects on renal function may worsen and lead to diabetic nephropathy [100]. However, bilateral renal denervation (BRD) has been shown to reduce AT1R expression and kidney damage, but BRD does not restore urinary sodium load (Una V) in STZ-induced diabetic rats [96]. In general, it seems that STZ-induced diabetes decreases the number of Ang II glomerular sites and increases the expression of AT1R in the cortical and outer medullary collecting duct and renal denervation refines these phenomena.

Increased activity of the renal sympathetic nervous system in thyroidectomy sheep embryos alters AT1R and AT2R mRNA and protein levels [102]. These receptors are involved in the development of the fetal kidney, and bilateral renal denervation normalizes changes in the expression of the Ang II receptor due to thyroidectomy. Therefore, thyroidectomy causes delayed maturation of renal RAS, and renal nerves modulate this condition [103].

### 1.6. Renal Denervation and Ang II Receptors in Other Organs

The sympathetic premotor neurons in RVLM (rostral ventrolateral medulla) and PVN (paraventricular nucleus of the hypothalamus) are the pathways that regulate renal function [104]. These brain nuclei are involved in cardiovascular function and have a high density of AT1R [105], through which Ang II creates its cardiovascular effects. Activation of AT1R in RVLM leads to increased blood pressure [106]. In unilateral ischemic renal denervation, reduction of blood pressure and decreased expression of AT1Rs and AT2Rs in the RVLM and PVN of the hypothalamus were observed in the hypertensive rats. However, their overall conclusion was that changed in Ang II receptor expression were due more to a reduction of blood pressure than to unilateral ischemic kidney denervation [76]. In addition, the upregulation of expression of the ACE2/Ang-(1–7)/Mas axis and downregulation of expression of the ACE/Ang II/AT1R axis were detected in the plasma and PVN in the renal denervation group of spontaneously hypertensive rats [107]. Dong et al. reported that two-week treatment of foot shock considerable increased systolic blood pressure, which was associated with increased angiotensinogen, renin, ACE1, and AT1aR mRNA and protein expression in the cerebral cortex and hypothalamus, and plasma concentrations of renin and Ang II were increased [108]. However, the systolic blood pressure was suppressed by renal denervation, and denervation reduced the major components of RAS not only in the circulatory system but also in the CNS [108].

The lamina terminalis (LT) and PVN are the sites that sense the peripheral signals generated in response to HF stress and lead to increased sympathetic activity, which exacerbates heart function [109, 110]. RAS also interacts with these nuclei synergistically to increase sympathetic activity. HF upregulates AT1R expression in LT and PVN [111, 112]. In the dog model of induced “rapid right ventricular pacing,” the effect of renal denervation on the progression of HF and the expression of AT1R transcripts and protein in the hypothalamus were studied. The level of AT1R protein in the hypothalamus of the HF control group and HF group with renal denervation increased significantly compared to the sham group [81]. In terms of expression of AT1R protein level in the hypothalamus, it was lower in the HF group subjected to renal denervation [81]. Li et al. showed that BRD improved isoproterenol-induced HF, by downregulating brain RAS, and renal denervation significantly reduces AT1R expression in both LT and PVN [112].

Finally, the effect of renal denervation on blood pressure and ventricular regeneration was investigated in rats, and it was found that a combination of myocardial infarction and renal denervation reduces the levels of norepinephrine, Ang II, cardiac Ang II, and AT1R [113].

In the aforementioned studies, the evaluation of receptors using different methods, such as ligand binding assay, real-time PCR, immunohistochemical assay, western blot, and ELISA has been used, which is mentioned in Table 1. It is necessary to give a brief explanation of an antibody-based method. Nonspecificity is a very common problem with antibodies, especially for those that recognize signaling proteins and receptors [114]. The results obtained with commercially available antibodies for RAS components need to be re-examined. A degree of specificity for antibodies is considered in their instruction sheets. These antibodies react with several additional unidentified proteins despite the fact that they identify the peptide antigen. Unfortunately, the use of any commercially available antibodies tested for AT1R and AT2R might result in false positive findings due to their lack of adequate specificity [115–120]. Commercially available AT1R and AT2R antibodies, for instance, recognize these receptors in cells lacking receptor gene expression and in animals with genetically altered AT1R or AT2R binding domains [116, 121]. Unfortunately, this issue affects all GPCR receptors as well as angiotensin II receptors, and it could even affect substances other than receptors [116]. The National Institutes of Health's specifications for antibody validation have been made public [121]. However, a more dependable method to date for examining the expression of AT1R and AT2R seems to be competitive radioligand binding and the measurement of mRNA expression [115, 122].

It should also be noted that, due to the very low endogenous levels of Ang II, measurement of this peptide is difficult. A broad range of Ang II levels in plasma and serum, ranging from 2 to 3500 pg/mL, has been reported in studies employing commercial ELISA. The fact that the ELISA's sensitivities are substantially lower than those claimed by the manufacturer may be the reason that it was unable to identify Ang II in plasma samples. Therefore, these tests do not provide a reliable assessment of Ang II in plasma samples [123].

## 2. Conclusion

The sympathetic system activity definitely affects RAS in the kidney. Renal denervation is associated with changes in the expression of Ang II receptors that may vary in normal conditions and diseases. So, interference in sympathetic activity with the RAS in the kidney is dependent on the expression and activity of the Ang II receptors (Table 1).

## Figures and Tables

**Table 1:** The effect of renal denervation on angiotensin II receptors in the kidney.

Model	Before renal denervation	Renal denervation	After renal denervation	Receptor assay	Reference
Normotensive rats Hydropenic rats (volume deficient state)	—	Unilateral	Increase of density, number, or affinity of glomerular Ang II receptors	Ligand binding assay	[4, 61, 66, 67]

Spontaneously hypertensive rats	—	Acute in left kidney	Increase of AT1R functionality in the renal vasculature	—	[65]

Spontaneously hypertensive rats	Decrease of vasoconstrictor response to vasoactive agents by carvedilol	Acute in left kidney	Increase the sensitivity of renal AT1R to the vasoactive agents	—	[7]

Spontaneously hypertensive rats	—	Bilateral (chemically)	Downregulate AT1R in plasma and PVN	Western blotting (antibodies against AT1R: No. ab124505, Abcam)	[107]

2K1C rats	Upregulate the expression of AT1R and AT2R mRNAs in ischemic kidney	Unilateral	Normalize the expression of AT1R and AT2R mRNAs in ischemic kidney	Quantitative real-time PCR	[76]

2K1C rats	Upregulate Ang II receptor expression in RVLM and PVN	Unilateral	Decrease of Ang II receptor expression in RVLM and PVN	Quantitative real-time PCR	[76]

Chronic foot shock-induced hypertension in rats	Increase of AT1aR mRNA and protein expression in cerebral cortex and hypothalamus	Bilateral	Decrease of AT1aR mRNA and protein expression in cerebral cortex and hypothalamus	AT1a mRNAs: real-time PCR Expressions of AT1a: western blotting (primary antibody: Santa Cruz Biotech, Inc.)	[108]

Chronic HF rabbit model	Increase of the proportion of AT1R to AT2R expression in renal cortex	Unilateral	Decrease of the AT1R expression and increase of the AT2R expression in the renal cortex	Antibody-based method (primary antibodies: AT1R and AT2R rabbit polyclonal antibody, 1:500, Santa Cruz)	[75, 83]

HF canine model	Upregulate mRNA expression and protein levels of AT1R hypothalamus	Catheter-based renal denervation	Decrease of mRNA expression and protein levels of AT1R in the hypothalamus	AT1 mRNAs: real-time PCR Protein expressions of AT1R: western blot	[81]

HF rats model	Upregulating mRNA expressions of AT1R in lamina terminalis and hypothalamic PVN	Bilateral	Reduced mRNA expressions of AT1R in lamina terminalis and hypothalamic PVN	Real-time PCR.	[112]

Ren-2 transgenic hypertensive rats with HF	No changes in renal Ang II receptor binding characteristics	Bilateral	Unchanged	Radioligand binding assay	[69]

Cardiomyopathy rats model	—	Bilateral	Downregulation of cardiac AT1R	Western blot (Abcam Inc, UK)	[74]

Cardiorenal syndrome rats model	Upregulation of AT1aR mRNA in the left ventricle and downregulation in the renal cortex	Bilateral	Ameliorate AT1aR mRNA in the left ventricle and downregulation in the renal cortex	Real-time PCR	[88]

MI rats model	—	Bilateral	Decrease of AT1R in heart	Western blot (primary antibody: AT1R, 1:1000; Abcam, UK)	[113]

Aortic regurgitation rats model	Increase of AT1aR mRNA in renal cortical tissue	Chronic unilateral	Suppressing the increased level of AT1aR mRNA	Real-time PCR	[90]

PKD rats model	Decrease of the mRNA expression level of AT1aR in kidney	Bilateral	No change	Real-time quantitative PCR	[95]

Diabetic rats model	Decrease in number of Ang II glomerular sites	Unilateral	Increase in the number of Ang II glomerular sites	Receptor-binding assay	[67]

Diabetic rats model	Increases in AT1R expression in the cortical and outer medullary collecting duct	Chronic bilateral	Decrease of the expression of AT1R in the cortical and outer medullary collecting duct	Immunohistochemistry assay (monoclonal AT1R: ab9391; 1:20, Abcam)	[96]

Sheep thyroidectomy fetuses model	Change AT1R and AT2R mRNA and protein levels in kidney	Bilateral	Regulate AT1R and AT2R receptor expression in kidney	AT1 and AT2 mRNA: RNase protection assay (RPA kit III; Ambion) AT1 and AT2 proteins: western blot	[103]

## Data Availability

Experimental data cannot be shared.

## References

[1] Johns E. J., Kopp U. C., DiBona G. F. (2011). Neural control of renal function. *Comprehensive Physiology*.

[2] Bader M., Ganten D. (2008). Update on tissue renin–angiotensin systems. *Journal of Molecular Medicine*.

[3] Pontes R. B., Girardi A. C., Nishi E. E., Campos R. R., Bergamaschi C. T. (2015). Crosstalk between the renal sympathetic nerve and intrarenal angiotensin II modulates proximal tubular sodium reabsorption. *Experimental Physiology*.

[4] Tucker B., Mundy C., Maciejewski A., Printz M., Ziegler M., Pelayo J. (1986). Changes in glomerular hemodynamic response to angiotensin II after subacute renal denervation in rats. *Journal of Clinical Investigation*.

[5] Osborn J. W., Tyshynsky R., Vulchanova L. (2021). Function of renal nerves in kidney physiology and pathophysiology. *Annual Review of Physiology*.

[6] Lohmeier T. E., Iliescu R. (2013). The sympathetic nervous system in obesity hypertension. *Current Hypertension Reports*.

[7] Abdulla M. H., Sattar M., Khan M. A., Abdullah N. A., Johns E. (2009). Influence of sympathetic and AT1‐receptor blockade on angiotensin II and adrenergic agonist‐induced renal vasoconstrictions in spontaneously hypertensive rats. *Acta Physiologica*.

[8] Clayton S. C., Mousa T. M., Zucker I. H. (2009). Sympathetic renal vasoconstriction in rabbits with pacing-induced heart failure is at1r dependent. https://www.ahajournals.org/doi/10.1161/circ.120.suppl_18.S718-c.

[9] Grassi G., Dell’Oro R., Quarti-Trevano F., Scopelliti F., Seravalle G., Paleari F. (2005). Neuroadrenergic and reflex abnormalities in patients with metabolic syndrome. *Diabetologia*.

[10] Sata Y., Head G. A., Denton K., May C. N., Schlaich M. P. (2018). Role of the sympathetic nervous system and its modulation in renal hypertension. *Frontiers of Medicine*.

[11] Fontes M. A. P., Marzano L. A. S., Silva C. C. (2020). Renal sympathetic denervation for resistant hypertension: where do we stand after more than a decade. *Brazilian Journal of Nephrology*.

[12] Converse R. L., Jacobsen T. N., Toto R. D., Jost C. M., Cosentino F., Fouad-Tarazi F. (1992). Sympathetic overactivity in patients with chronic renal failure. *New England Journal of Medicine*.

[13] Moss N. G. (1982). Renal function and renal afferent and efferent nerve activity. *American Journal of Physiology—Renal Physiology*.

[14] Mulder J., Hökfelt T., Knuepfer M. M., Kopp U. C. (2013). Renal sensory and sympathetic nerves reinnervate the kidney in a similar time-dependent fashion after renal denervation in rats. *American Journal of Physiology—Regulatory, Integrative and Comparative Physiology*.

[15] Carey R. M. (2015). The intrarenal renin-angiotensin system in hypertension. *Advances in Chronic Kidney Disease*.

[16] Li X. C., Zhuo J. L. (2008). Intracellular ANG II directly induces in vitro transcription of TGF-*β*1, MCP-1, and NHE-3 mRNAs in isolated rat renal cortical nuclei via activation of nuclear AT1a receptors. *American Journal of Physiology—Cell Physiology*.

[17] Savoia C., Burger D., Nishigaki N., Montezano A., Touyz R. M. (2011). Angiotensin II and the vascular phenotype in hypertension. *Expert Reviews in Molecular Medicine*.

[18] Mizuiri S., Ohashi Y. (2015). ACE and ACE2 in kidney disease. *World Journal of Nephrology*.

[19] Varagic J., Ahmad S., Nagata S., Ferrario C. M. (2014). ACE2: angiotensin II/angiotensin-(1–7) balance in cardiac and renal injury. *Current Hypertension Reports*.

[20] Cantero-Navarro E., Fernández-Fernández B., Ramos A. M., Rayego-Mateos S., Rodrigues-Diez R. R., Sánchez-Niño M. D. (2021). Renin-angiotensin system and inflammation update. *Molecular and Cellular Endocrinology*.

[21] Kawai T., Forrester S. J., O’Brien S., Baggett A., Rizzo V., Eguchi S. (2017). AT1 receptor signaling pathways in the cardiovascular system. *Pharmacological Research*.

[22] Guo D. F., Sun Y. L., Hamet P., Inagami T. (2001). The angiotensin II type 1 receptor and receptor-associated proteins. *Cell Research*.

[23] Allen A. M., Zhuo J., Mendelsohn F. A. (2000). Localization and function of angiotensin AT1 receptors. *American Journal of Hypertension*.

[24] Smith R. D., Timmermans P. B. (1994). Human angiotensin receptor subtypes. *Current Opinion in Nephrology and Hypertension*.

[25] Konishi H., Kuroda Si, Inada Y., Fujisawa Y. (1994). Novel subtype of human angiotensin II type 1 receptor: cDNA cloning and expression. *Biochemical and Biophysical Research Communications*.

[26] Siragy H. M. (2004). AT1 and AT2 receptor in the kidney: role in health and disease. *Seminars in Nephrology*.

[27] Lai K. N., Chan L. Y., Tang S. C., Tsang A. W., Li F. F., Lam M. F. (2004). Mesangial expression of angiotensin II receptor in IgA nephropathy and its regulation by polymeric IgA1. *Kidney International*.

[28] Gonzalez A. A., Luffman C., Bourgeois C. R., Vio C. P., Prieto M. C. (2013). Angiotensin II–independent upregulation of Cyclooxygenase-2 by activation of the (pro) renin receptor in rat renal inner medullary cells. *Hypertension*.

[29] Bivol L. M., Vågnes Ø. B., Iversen B. M. (2005). The renal vascular response to ANG II injection is reduced in the nonclipped kidney of two-kidney, one-clip hypertension. *American Journal of Physiology—Renal Physiology*.

[30] Siragy H. M. (2002). Angiotensin receptor blockers: how important is selectivity?. *American Journal of Hypertension*.

[31] Siragy H. M. (2000). AT1 and AT2 receptors in the kidney: role in disease and treatment. *American Journal of Kidney Diseases*.

[32] Rozenfeld R., Gupta A., Gagnidze K., Lim M. P., Gomes I., Lee‐Ramos D. (2011). AT1R–CB1R heteromerization reveals a new mechanism for the pathogenic properties of angiotensin II. *The EMBO Journal*.

[33] Raizada V., Skipper B., Luo W., Griffith J. (2007). Intracardiac and intrarenal renin-angiotensin systems: mechanisms of cardiovascular and renal effects. *Journal of Investigative Medicine*.

[34] Castrop H., Höcherl K., Kurtz A., Schweda F., Todorov V., Wagner C. (2010). Physiology of kidney renin. *Physiological Reviews*.

[35] Gonzalez A. A., Liu L., Lara L. S., Bourgeois C. R., Ibaceta-Gonzalez C., Salinas-Parra N. (2015). PKC-*α*-dependent augmentation of cAMP and CREB phosphorylation mediates the angiotensin II stimulation of renin in the collecting duct. *American Journal of Physiology - Renal Physiology*.

[36] Li X. C., Hopfer U., Zhuo J. L. (2009). AT1 receptor-mediated uptake of angiotensin II and NHE-3 expression in proximal tubule cells through a microtubule-dependent endocytic pathway. *American Journal of Physiology—Renal Physiology*.

[37] Arima S., Kohagura K., Abe M., Ito S. (2001). Mechanisms that control glomerular hemodynamics. *Clinical and Experimental Nephrology*.

[38] Lins B. B., Casare F. A. M., Fontenele F. F., Gonçalves G. L., Oliveira-Souza M. (2021). Long-term angiotensin II infusion induces oxidative and endoplasmic reticulum stress and modulates Na+ transporters through the nephron. *Frontiers in Physiology*.

[39] Berry C., Touyz R., Dominiczak A., Webb R. C., Johns D. (2001). Angiotensin receptors: signaling, vascular pathophysiology, and interactions with ceramide. *American Journal of Physiology—Heart and Circulatory Physiology*.

[40] Berry C., Hamilton C. A., Brosnan M. J., Magill F. G., Berg G. A., McMurray J. J. (2000). Investigation into the sources of superoxide in human blood vessels: angiotensin II increases superoxide production in human internal mammary arteries. *Circulation*.

[41] Vaziri N. D., Bai Y., Ni Z., Quiroz Y., Pandian R., Rodriguez-Iturbe B. (2007). Intra-renal angiotensin II/AT1 receptor, oxidative stress, inflammation, and progressive injury in renal mass reduction. *Journal of Pharmacology and Experimental Therapeutics*.

[42] Carey R. M., Wang Z.-Q., Siragy H. M. (2000). Role of the angiotensin type 2 receptor in the regulation of blood pressure and renal function. *Hypertension*.

[43] Vinturache A. E., Smith F. G. (2018). Glomerular and tubular effects of nitric oxide (NO) are regulated by angiotensin II (Ang II) in an age-dependent manner through activation of both angiotensin receptors (AT1Rs and AT2Rs) in conscious lambs. *Pfluegers Archiv European Journal of Physiology*.

[44] Arima S., Ito S. (2000). Angiotensin II type 2 receptors in the kidney: evidence for endothelial-cell-mediated renal vasodilatation. *Nephrology Dialysis Transplantation*.

[45] Gross V., Schunck W.-H., Honeck H., Milia A. F., Kärgel E., Walther T. (2000). Inhibition of pressure natriuresis in mice lacking the AT2 receptor. *Kidney International*.

[46] Carey R. M., Padia S. H. (2013). Role of angiotensin AT (2) receptors in natriuresis: intrarenal mechanisms and therapeutic potential. *Clinical and Experimental Pharmacology and Physiology*.

[47] Chappell M. C. (2012). The non-classical renin-angiotensin system and renal function. *Comprehensive Physiology*.

[48] Quadri S. S., Culver S. A., Li C., Siragy H. M. (2016). Interaction of the renin angiotensin and cox systems in the kidney. *Frontiers in Bioscience*.

[49] Siragy H. M. (2010). The angiotensin II type 2 receptor and the kidney. *Journal of the Renin-Angiotensin-Aldosterone System*.

[50] Carey R. M., Wang Z.-Q., Siragy H. M. (1999). Novel actions of angiotensin II via its renal type-2 (AT 2) receptor. *Current Hypertension Reports*.

[51] Miller A. J., Arnold A. C. (2019). The renin–angiotensin system in cardiovascular autonomic control: recent developments and clinical implications. *Clinical Autonomic Research*.

[52] Ferrario C. M., Ahmad S., Joyner J., Varagic J. (2010). Advances in the renin angiotensin system: focus on angiotensin-converting enzyme 2 and angiotensin-(1–7). *Advances in Pharmacology*.

[53] Liao W., Wu J. (2021). The ACE2/Ang (1–7)/MasR axis as an emerging target for antihypertensive peptides. *Critical Reviews in Food Science and Nutrition*.

[54] Dai H., Jiang L., Xiao Z., Guang X. (2015). ACE2–angiotensin-(1-7)–Mas axis might be a promising therapeutic target for pulmonary arterial hypertension. *Nature Reviews Cardiology*.

[55] Rodrigues Prestes T. R., Rocha N. P., Miranda A. S., Teixeira A. L., Simoes-e-Silva A. C. (2017). The anti-inflammatory potential of ACE2/angiotensin-(1-7)/Mas receptor axis: evidence from basic and clinical research. *Current Drug Targets*.

[56] Moon J.-Y. (2013). Recent update of renin-angiotensin-aldosterone system in the pathogenesis of hypertension. Electrolytes & Blood Pressure. *BPN*.

[57] Azushima K., Morisawa N., Tamura K., Nishiyama A. (2020). Recent research advances in renin-angiotensin-aldosterone system receptors. *Current Hypertension Reports*.

[58] Dibona G. F. (2001). Peripheral and central interactions between the renin‐angiotensin system and the renal sympathetic nerves in control of renal function. *Annals of the New York Academy of Sciences*.

[59] Dibona G. F. (2000). Nervous kidney: interaction between renal sympathetic nerves and the renin-angiotensin system in the control of renal function. *Hypertension*.

[60] Salman I., Sattar M., Ameer O., Abdullah N., Yam M., Salman H. (2010). Role of norepinephrine & angiotensin II in the neural control of renal sodium and water handling in spontaneously hypertensive rats. *Indian Journal of Medical Research*.

[61] Sahlgren B., Eklöf A. C., Aperia A. (1992). Regulation of glomerular angiotensin II receptor densities in renovascular hypertension: response to reduced sympathetic and vasopressin influence. *Acta Physiologica Scandinavica*.

[62] Wagner C., Kees F., Krämer B. K., Kurtz A. (1997). Role of sympathetic nerves fo the stimulation of the renin system by angiotensin II receptor blockade. *Journal of Hypertension*.

[63] Iliescu R., Lohmeier T. E., Tudorancea I., Laffin L., Bakris G. L. (2015). Renal denervation for the treatment of resistant hypertension: review and clinical perspective. *American Journal of Physiology - Renal Physiology*.

[64] Ichihara A., Inscho E. W., Imig J. D., Michel R. E., Navar L. G. (1997). Role of renal nerves in afferent arteriolar reactivity in angiotensin-induced hypertension. *Hypertension*.

[65] Abdulla M., Sattar M., Salman I., Abdullah N., Ameer O., Khan M. A. (2008). Effect of acute unilateral renal denervation on renal hemodynamics in spontaneously hypertensive rats. *Autonomic and Autacoid Pharmacology*.

[66] Pelayo J. C., Blantz R. C. (1984). Analysis of renal denervation in the hydropenic rat: interactions with angiotensin II. *American Journal of Physiology—Renal Physiology*.

[67] Wilkes B. M., Pion I., Sollott S., Michaels S., Kiesel G. (1988). Intrarenal renin-angiotensin system modulates glomerular angiotensin receptors in the rat. *American Journal of Physiology—Renal Physiology*.

[68] Cheng H.-F., Becker B. N., Burns K., Harris R. (1995). Angiotensin II upregulates type-1 angiotensin II receptors in renal proximal tubule. *Journal of Clinical Investigation*.

[69] Honetschlägerová Z., Hejnová L., Novotný J., Marek A., Červenka L. (2021). Effects of renal denervation on the enhanced renal vascular responsiveness to angiotensin II in high-output heart failure: angiotensin II receptor binding assessment and functional studies in ren-2 transgenic hypertensive rats. *Biomedicines*.

[70] Schlaich M. P., Krum H., Sobotka P. A., Esler M. D. (2011). Renal denervation and hypertension. *American Journal of Hypertension*.

[71] Sobotka P. A., Krum H., Böhm M., Francis D. P., Schlaich M. P. (2012). The role of renal denervation in the treatment of heart failure. *Current Cardiology Reports*.

[72] Frame A. A., Carmichael C. Y., Wainford R. D. (2016). Renal afferents. *Current Hypertension Reports*.

[73] Huggett R. J., Scott E. M., Gilbey S. G., Stoker J. B., Mackintosh A. F., Mary D. A. (2003). Impact of type 2 diabetes mellitus on sympathetic neural mechanisms in hypertension. *Circulation*.

[74] Liu Q., Zhang Q., Wang K., Wang S., Lu D., Li Z. (2015). Renal denervation findings on cardiac and renal fibrosis in rats with isoproterenol induced cardiomyopathy. *Scientific Reports*.

[75] Clayton S. C., Haack K. K., Zucker I. H. (2011). Renal denervation modulates angiotensin receptor expression in the renal cortex of rabbits with chronic heart failure. *American Journal of Physiology - Renal Physiology*.

[76] Nishi E. E., Lopes N. R., Gomes G. N., Perry J. C., Sato A., Naffah-Mazzacoratti M. G. (2019). Renal denervation reduces sympathetic overactivation, brain oxidative stress, and renal injury in rats with renovascular hypertension independent of its effects on reducing blood pressure. *Hypertension Research*.

[77] Inoue N., Nagao K., Hirata J., Wang Y.-M., Yanagita T. (2004). Conjugated linoleic acid prevents the development of essential hypertension in spontaneously hypertensive rats. *Biochemical and Biophysical Research Communications*.

[78] Doggrell S. A., Brown L. (1998). Rat models of hypertension, cardiac hypertrophy and failure. *Cardiovascular Research*.

[79] Krum H., Schlaich M. P., Sobotka P. A., Böhm M., Mahfoud F., Rocha-Singh K. (2014). Percutaneous renal denervation in patients with treatment-resistant hypertension: final 3-year report of the Symplicity HTN-1 study. *The Lancet*.

[80] Esler M., Guo L. (2017). The future of renal denervation. *Autonomic Neuroscience*.

[81] Chen W.-J., Liu H., Wang Z.-H., Liu C., Fan J.-Q., Wang Z.-L. (2020). The impact of renal denervation on the progression of heart failure in a canine model induced by right ventricular rapid pacing. *Frontiers in Physiology*.

[82] DiBona G. F. (2005). Dynamic analysis of patterns of renal sympathetic nerve activity: implications for renal function. *Experimental Physiology*.

[83] Clayton S. C., Curry P. L., Li Y., Zucker I. H. (2008). *Exercise Training and Renal Denervation Attenuate the Expression of Angiotensin II Type 1 and 2 Receptors in Rabbits with Chronic Heart Failure*.

[84] Wang T.-T., Wu X.-H., Zhang S.-L., Chan J. S. (1998). Molecular mechanism (s) of action of norepinephrine on the expression of the angiotensinogen gene in opossum kidney cells. *Kidney International*.

[85] Vikhorev P. G., Vikhoreva N. N. (2018). Cardiomyopathies and related changes in contractility of human heart muscle. *International Journal of Molecular Sciences*.

[86] Braam B., Joles J. A., Danishwar A. H., Gaillard C. A. (2014). Cardiorenal syndrome—current understanding and future perspectives. *Nature Reviews Nephrology*.

[87] Bongartz L. G., Cramer M. J., Doevendans P. A., Joles J. A., Braam B. (2005). The severe cardiorenal syndrome: Guyton revisited. *European Heart Journal*.

[88] Eriguchi M., Tsuruya K., Haruyama N., Yamada S., Tanaka S., Suehiro T. (2015). Renal denervation has blood pressure–independent protective effects on kidney and heart in a rat model of chronic kidney disease. *Kidney International*.

[89] Bekeredjian R., Grayburn P. A. (2005). Valvular heart disease: aortic regurgitation. *Circulation*.

[90] Rafiq K., Noma T., Fujisawa Y., Ishihara Y., Arai Y., Nabi A. N. (2012). Renal sympathetic denervation suppresses de novo podocyte injury and albuminuria in rats with aortic regurgitation. *Circulation*.

[91] Wüthrich R. P., Mei C. (2014). Pharmacological management of polycystic kidney disease. *Expert Opinion on Pharmacotherapy*.

[92] Helal I., Reed B., Mettler P., Mc Fann K., Tkachenko O., Yan X.-D. (2012). Prevalence of cardiovascular events in patients with autosomal dominant polycystic kidney disease. *American Journal of Nephrology*.

[93] Ecder T., Schrier R. W. (2001). Hypertension in autosomal-dominant polycystic kidney disease: early occurrence and unique aspects. *Journal of the American Society of Nephrology*.

[94] Goto M., Hoxha N., Osman R., Dell K. M. (2010). The renin-angiotensin system and hypertension in autosomal recessive polycystic kidney disease. *Pediatric Nephrology*.

[95] Li S., Hildreth C. M., Rahman A. A., Barton S. A., Wyse B. F., Lim C. K. (2021). Renal denervation does not affect hypertension or the renin-angiotensin system in a rodent model of juvenile-onset polycystic kidney disease: clinical implications. *Scientific Reports*.

[96] Yao Y., Fomison-Nurse I. C., Harrison J. C., Walker R. J., Davis G., Sammut I. A. (2014). Chronic bilateral renal denervation attenuates renal injury in a transgenic rat model of diabetic nephropathy. *American Journal of Physiology—Renal Physiology*.

[97] Ballermann B., Skorecki K., Brenner B. (1984). Reduced glomerular angiotensin II receptor density in early untreated diabetes mellitus in the rat. *American Journal of Physiology—Renal Physiology*.

[98] Hostetter T. H., Troy J. L., Brenner B. M. (1981). Glomerular hemodynamics in experimental diabetes mellitus. *Kidney International*.

[99] Wilkes B. M. (1987). Reduced glomerular angiotensin II receptor density in diabetes mellitus in the rat: time course and mechanism. *Endocrinology*.

[100] Harrison-Bernard L. M., Imig J. D., Carmines P. K. (2002). Renal AT1 receptor protein expression during the early stage of diabetes mellitus. *International Journal of Experimental Diabetes Research*.

[101] Kobori H., Kamiyama M., Harrison-Bernard L. M., Navar L. G. (2013). Cardinal role of the intrarenal renin-angiotensin system in the pathogenesis of diabetic nephropathy. *Journal of Investigative Medicine*.

[102] Chen K., Carey L. C., Valego N. K., Liu J., Rose J. C. (2005). Thyroid hormone modulates renin and ANG II receptor expression in fetal sheep. *American Journal of Physiology—Regulatory, Integrative and Comparative Physiology*.

[103] Chen K., Carey L. C., Valego N. K., Liu J., Rose J. C. (2006). Combined thyroidectomy and renal denervation suppress renin expression and secretion in fetal sheep. *Journal of the Society for Gynecologic Investigation: JSGI*.

[104] Nishi E. E., Bergamaschi C. T., Campos R. R. (2015). The crosstalk between the kidney and the central nervous system: the role of renal nerves in blood pressure regulation. *Experimental Physiology*.

[105] Jiang N., Shi P., Li H., Lu S., Braseth L., Cuadra A. E. (2009). Phosphate-activated glutaminase-containing neurons in the rat paraventricular nucleus express angiotensin type 1 receptors. *Hypertension*.

[106] de Oliveira-Sales E. B., Nishi E. E., Boim M. A., Dolnikoff M. S., Bergamaschi C. T., Campos R. R. (2010). Upregulation of AT1R and iNOS in the rostral ventrolateral medulla (RVLM) is essential for the sympathetic hyperactivity and hypertension in the 2K-1C Wistar rat model. *American Journal of Hypertension*.

[107] Han W., Wang M., Zhai X., Gan Q., Guan S., Qu X. (2020). Chemical renal denervation-induced upregulation of the ACE2/Ang (1-7)/Mas axis attenuates blood pressure elevation in spontaneously hypertensive rats. *Clinical and Experimental Hypertension*.

[108] Dong T., Chen J.-W., Tian L.-L., Wang L.-H., Jiang R.-D., Zhang Z. (2015). Role of the renin-angiotensin system, renal sympathetic nerve system, and oxidative stress in chronic foot shock-induced hypertension in rats. *International Journal of Biological Sciences*.

[109] Felder R. B., Francis J., Zhang Z.-H., Wei S.-G., Weiss R. M., Johnson A. K. (2003). Heart failure and the brain: new perspectives. *American Journal of Physiology - Regulatory, Integrative and Comparative Physiology*.

[110] Johnson A. K., Gross P. M. (1993). Sensory circumventricular organs and brain homeostatic pathways. *The FASEB Journal*.

[111] Kang Y.-M., Ma Y., Elks C., Zheng J.-P., Yang Z.-M., Francis J. (2008). Cross-talk between cytokines and renin–angiotensin in hypothalamic paraventricular nucleus in heart failure: role of nuclear factor-*κ*B. *Cardiovascular Research*.

[112] Li J.-D., Cheng A.-Y., Huo Y.-L., Fan J., Zhang Y.-P., Fang Z.-Q. (2016). Bilateral renal denervation ameliorates isoproterenol-induced heart failure through downregulation of the brain renin-angiotensin system and inflammation in rat. *Oxidative Medicine and Cellular Longevity*.

[113] Liu S., Wang G., Ding X., Paz M. A., Xu X., Dong F. (2017). Renal sympathetic denervation raises blood pressure and attenuates ventricular remodeling in rats with myocardial infarction. *International Journal of Clinical and Experimental Medicine*.

[114] Gautron L. (2019). On the necessity of validating antibodies in the immunohistochemistry literature. *Frontiers in Neuroanatomy*.

[115] Hafko R., Villapol S., Nostramo R., Symes A., Sabban E. L., Inagami T. (2013). Commercially available angiotensin II At2 receptor antibodies are nonspecific. *PLoS One*.

[116] Saavedra J. (2017). Beneficial effects of Angiotensin II receptor blockers in brain disorders. *Pharmacological Research*.

[117] Benicky J., Hafko R., Sanchez-Lemus E., Aguilera G., Saavedra J. M. (2012). Six commercially available angiotensin II AT1 receptor antibodies are non-specific. *Cellular and Molecular Neurobiology*.

[118] Forrester S. J., Booz G. W., Sigmund C. D., Coffman T. M., Kawai T., Rizzo V. (2018). Angiotensin II signal transduction: an update on mechanisms of physiology and pathophysiology. *Physiological Reviews*.

[119] Siljee S., Milne B., Brasch H. D., Bockett N., Patel J., Davis P. F. (2021). Expression of components of the renin-angiotensin system by cancer stem cells in renal clear cell carcinoma. *Biomolecules*.

[120] Siljee S., Buchanan O., Brasch H. D., Bockett N., Patel J., Paterson E. (2021). Cancer stem cells in metastatic head and neck cutaneous squamous cell carcinoma express components of the renin-angiotensin system. *Cells*.

[121] Saavedra J. M. (2021). Angiotensin receptor blockers are not just for hypertension anymore. *Physiology*.

[122] Xu X., He H., Hu S., Han J.-B., Huang L., Xu J.-Y. (2017). Ang II-AT2R increases mesenchymal stem cell migration by signaling through the FAK and RhoA/Cdc42 pathways in vitro. *Stem Cell Research and Therapy*.

[123] Chappell M. C., Pirro N. T., South A. M., Gwathmey T. M. (2021). Concerns on the specificity of commercial ELISAs for the measurement of angiotensin (1–7) and angiotensin II in human plasma. *Hypertension*.

